# Binding Free Energy (BFE) Calculations and Quantitative Structure–Activity Relationship (QSAR) Analysis of *Schistosoma mansoni* Histone Deacetylase 8 (*sm*HDAC8) Inhibitors

**DOI:** 10.3390/molecules26092584

**Published:** 2021-04-28

**Authors:** Conrad V. Simoben, Ehab Ghazy, Patrik Zeyen, Salma Darwish, Matthias Schmidt, Christophe Romier, Dina Robaa, Wolfgang Sippl

**Affiliations:** 1 Institute of Pharmacy, Martin-Luther University of Halle-Wittenberg, 06120 Halle (Saale), Germany; veranso.conrad@gmail.com (C.V.S.); ehab.ghazy@pharmazie.uni-halle.de (E.G.); patrik.zeyen@pharmazie.uni-halle.de (P.Z.); salma.darwish@pharmazie.uni-halle.de (S.D.); matthias.schmidt@pharmazie.uni-halle.de (M.S.); dina.robaa@pharmazie.uni-halle.de (D.R.); 2 Département de Biologie Structurale Intégrative, Institut de Génétique et Biologie Moléculaire et Cellulaire (IGBMC), University of Strasbourg, CNRS, INSERM, 1 rue Laurent Fries, CEDEX, 67404 Illkirch, France; romier@igbmc.fr

**Keywords:** binding free energy calculations, *Schistosoma mansoni*, histone deacetylase inhibitors, quantitative structure–activity relationship (QSAR)

## Abstract

Histone-modifying proteins have been identified as promising targets to treat several diseases including cancer and parasitic ailments. *In silico* methods have been incorporated within a variety of drug discovery programs to facilitate the identification and development of novel lead compounds. In this study, we explore the binding modes of a series of benzhydroxamates derivatives developed as histone deacetylase inhibitors of *Schistosoma mansoni* histone deacetylase (*sm*HDAC) using molecular docking and binding free energy (BFE) calculations. The developed docking protocol was able to correctly reproduce the experimentally established binding modes of resolved *sm*HDAC8–inhibitor complexes. However, as has been reported in former studies, the obtained docking scores weakly correlate with the experimentally determined activity of the studied inhibitors. Thus, the obtained docking poses were refined and rescored using the Amber software. From the computed protein–inhibitor BFE, different quantitative structure–activity relationship (QSAR) models could be developed and validated using several cross-validation techniques. Some of the generated QSAR models with good correlation could explain up to ~73% variance in activity within the studied training set molecules. The best performing models were subsequently tested on an external test set of newly designed and synthesized analogs. In vitro testing showed a good correlation between the predicted and experimentally observed IC_50_ values. Thus, the generated models can be considered as interesting tools for the identification of novel *sm*HDAC8 inhibitors.

## 1. Introduction

Neglected parasitic diseases have been responsible for morbidity and mortality of hundreds of millions of humans in underprivileged communities especially in parts of the Middle East, South America, Southeast Asia and, particularly, in sub-Saharan Africa for over many decades [1]. Amongst these neglected parasitic diseases is schistosomiasis (bilharzia), which is a common intravascular parasitic infection in humans caused by *Schistosoma* spp. [2]. Despite being a preventable illness, chronic infection is associated with long-term undernutrition, anaemia, organ scarring and fibrosis, resulting in disabling patient symptoms [2,3,4]. According to the World Health Organization (WHO), an estimated 206.5 million people required preventive treatment for schistosomiasis, out of which more than 89 million people were reported to have been treated [3,4]. Several detailed studies/reviews explaining the epidemiology of schistosomiasis have been published [1,2,3,4,5]. In recent years, national and international programs have been implemented in regular drug administration to control or prevent *Schistosoma* infections. Interestingly, there has been a decrease in the number of infected individuals. However, with no available vaccine, new small molecule inhibitors to treat the disease are needed. In addition, drug resistance problems have been reported for the current drug of choice, praziquantel [2,6,7,8,9,10].

A promising strategy to treat schistosomiasis amongst other parasitic diseases is to target the epigenome of the parasite [11,12,13]. Histone modifying proteins have thus emerged as potential targets to modulate the epigenome of this parasite in the hope to treat this parasitic ailment. Among these histone modifying proteins are histone deacetylases (HDACs, sometimes also referred to as lysine deacetylases, KDACs), which function by regulating the deacetylation of histone lysine residues as a part of DNA transcriptional regulation [14]. An upset of this balance can lead to hypoacetylation or hyperacetylation leading to the manifestation of diseases such as cancer, inflammation, etc. Conventionally, there are 18 known human HDAC isoforms divided into four major classes (classes I, II, III and IV) depending on their homology to yeast (*Saccharomyces cerevisiae*) HDACs [15]. While class I (HDAC1, 2, 3 and 8), class IIa (HDAC4, 5 and 7), class IIb (HDAC6 and 10) and class IV (HDAC11) are Zn^2+^-dependent for their deacetylase activity, class III (Sirtuins 1–7) are nicotinamide adenine dinucleotide (NAD^+^)-dependent protein deacetylases [15,16,17,18,19,20]. Although HDACs have recently emerged as potential targets to treat cancer and parasitic diseases amongst others, the biology behind the effect of HDAC inhibition on these diseases is still not fully understood [21,22,23,24,25].

For over three decades, HDACs have been targeted for various cancer treatments which have resulted in the approval of five HDAC inhibitors with several others in clinical trials for the treatment of several types of cancer [26,27,28,29,30,31,32,33,34,35,36,37,38]. Most of the designed, developed and reported HDAC inhibitors have a general structural pharmacophore consisting of three features—the zinc-binding group (ZBG), the linker and the capping group (cap) [39,40,41,42]. Additionally, HDAC inhibitors bearing a hydroxamate group chelating the catalytic zinc ion are the most investigated. Due to the partially conserved nature across the active site in all HDACs with a substrate binding channel and the relatively similar pharmacophoric features of the reported HDAC inhibitors, the desired isoform(s) selectivity represents a challenge [27,43,44]. Many of the published hydroxamate-type inhibitors inhibit most HDAC isoforms, which limits their use as pharmacological tools and may lead to side effects in the clinic. Resolved crystal structures also reveal that a majority of the hydroxamate derivatives, as well as some other reported zinc chelating groups are able to coordinate the catalytic zinc ion in a bidentate fashion [45,46]. Nevertheless, some recently released crystal structures of HDAC6 isoform shows that some HDAC inhibitors coordinate the catalytic zinc ion in a mono-dentate fashion [47,48]. In addition to the zinc ion coordination, HDAC inhibitors are further stabilized by hydrogen bonds, mostly with the two conserved histidine residues and the catalytic tyrosine residue, as well as van der Waals and π–π-interaction(s) amongst others.

Several studies have explored the major pharmacophoric features and interactions observed with HDAC inhibitors for the development of new drug candidates against schistosomiasis to target the parasite’s epigenome [49]. To reduce the potential side effects of the new molecules, specific targeting of schistosomal HDACs is very important [50]. Interestingly, the human orthologue of *S. mansoni* HDAC8 (*sm*HDAC8), human HDAC8 (*hs*HDAC8), is less abundant in humans than other class I HDACs (HDAC1 and 3) except in some tumor cells where it is up-regulated [51]. Thus, the development of small-molecule *sm*HDAC8 inhibitors represents a promising approach for the treatment of schistosomiasis. We have previously reported on a series of *sm*HDAC8 inhibitors through virtual screening-based identification and structure-guided optimization [49,52,53,54]. Application of computational methods to aid our quest for new *sm*HDAC8 inhibitors led to the identification of several potential scaffolds [52,54]. Amongst the identified scaffolds, the *m*-substituted benzhydroxamates were identified as promising lead structures and further structural optimization studies of this series led to potent *sm*HDAC8 inhibitors [54]. The developed benzhydroxamates exerted interesting selectivity over relevant human HDAC isoforms (HDAC1, 3 and 6; in some cases also HDAC8) and induced apoptosis and mortality of schistosomes in cellular assays. Selectivity of these molecules has been attributed to the possibility of these molecules to target the side pocket present in HDACs 6 and 8 as stipulated in the binding modes from docking procedures and resolved crystal structures. Furthermore, some selected compounds displayed significant dose-dependent killing of the schistosome larvae and markedly impaired egg laying of adult worm pairs maintained in culture.

In this study, we first set to generate structure-based QSAR models that were able to explain variations in the determined *sm*HDAC8 IC_50_ values. The validated models were subsequently used to predict the activities of some newly designed benzhydroxamate derivatives as *sm*HDAC8 inhibitors and to propose the most promising ones for synthesis and biological evaluation.

## 2. Results and Discussion

### 2.1. Diversity Analysis of Dataset(s)

**Figure 1:**
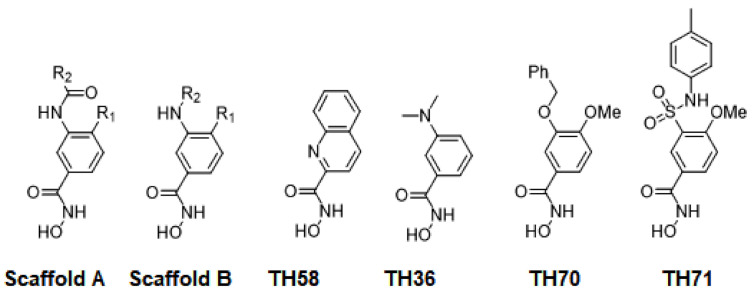
Training set molecules.

**Figure 2:**
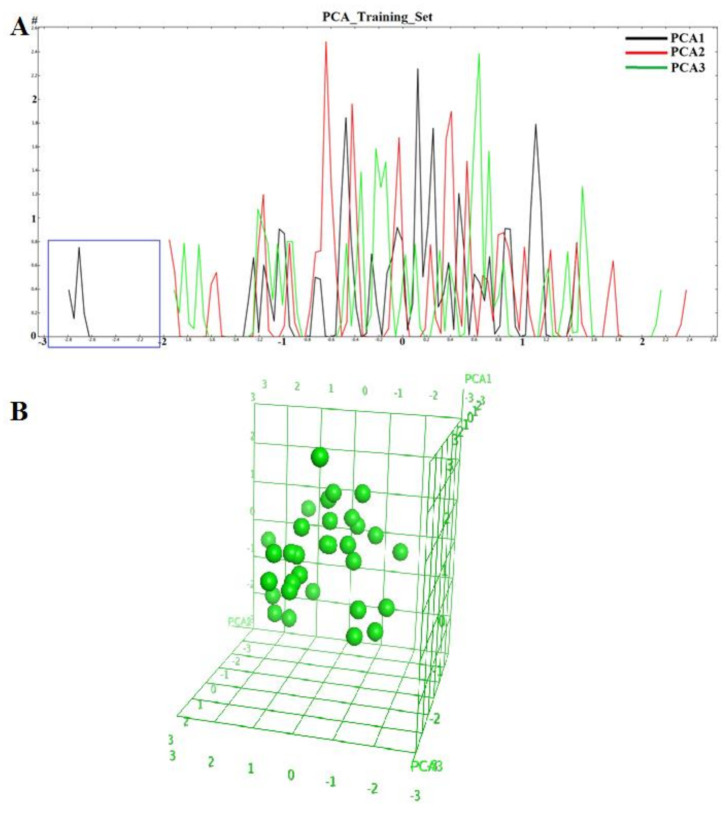
(**A**) 2D plots to visualize the variation of the three most important computed principal components for the training set (Blue box represent the outlier TH58) and (**B**) 3D visualization of the chemical space occupied by the training set molecules using the first three PCAs.

In order to develop a robust QSAR model, accuracy and precision of the biological data are very important. Thus, the selection of molecules to build the model holds a critical place in the development process. A critical aspect of the selection is to focus on a particular series/chemical space of molecules while covering a reasonable biological activity range. In this study, benzhydroxamic acids—an important class of HDAC inhibitors that we recently developed as inhibitors of the HDAC8 isoform (see the Materials and Methods section) constituted the training set molecules. These molecules in the training set can be generally characterized by the scaffolds depicted in Figure 1, were R1 (at the para position) represents small groups like hydrogen, halogen, methyl, methoxy, while R2 (meta position) represents aromatic substitutions. Reported studies show that this class of molecule has well-established interactions with amino acids residues within the binding site of *sm*HDAC8. For instance, the hydroxamate function (ZBG) coordinates the catalytic Zn^2+^ ions while additionally forming hydrogen-bond interactions with Tyr304, His140 and/or His141. On the other hand, the carbonyl functional group (in the case of the amides-scaffold) forms a hydrogen bond with Lys20 while the amide/amine H atom is observed to form hydrogen bond with His292. We began our studies by analyzing the diversity of the selected compounds (Figure 1 and Table 1 for information about the selected compounds) using principal component analysis (PCA). The applicability domain of the studied dataset can be used to define a model’s limitations. Figure 2 shows the two-dimensional (2D) plot of the variations (Figure 2A) and the 3D representation (Figure 2B) of the three most important components (PCA1, PCA2 and PCA3) of the computed descriptors (a_heavy, b_1rotN, b_single, lip_acc, lip_don, mr, PEOE_VSA_POL, TPSA, h_logD and PEOE_VSA_FPPOS) for the training dataset. Distribution of the three most important PCAs after linear transformation using PCA showed that the molecules used in this study were distributed homogeneously within the PCA space. Interestingly, analysis of the three most important principal components of the molecular descriptors space could explain approximately 100% covariation of the global information of the original space.

### 2.2. Molecular Docking

At the beginning of this study several co-crystallized protein–ligand structures of *sm*HDAC8 were known and available in the protein databank (https://www.rcsb.org/ (Accessed 16 January 2020)). The existence of these deposited complexes allowed the restriction of the molecules to be docked in the binding site only and the prior knowledge of reported ligand–target interactions/pose helped in selecting poses that are closest to the experimentally reported pose. As mentioned above, 25 crystal structures of *sm*HDAC8 in complex with various inhibitors were studied in the current work. The selection of the most suitable crystal structure for the docking protocol was based on re- and cross-docking studies.

**Figure 3:**
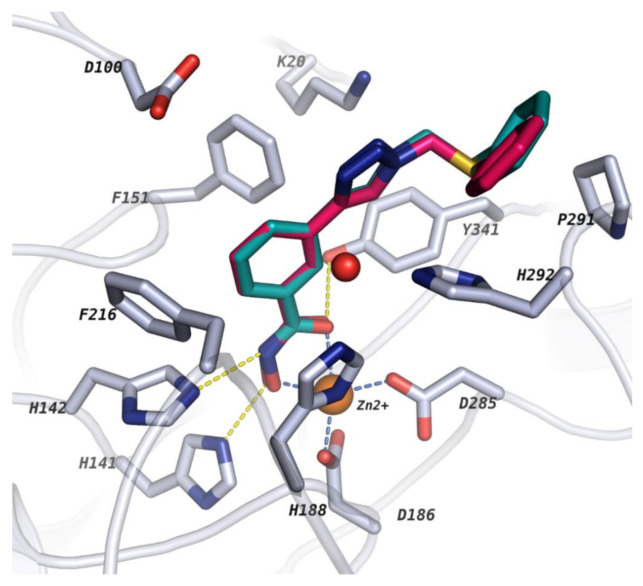
Re-docked pose in PDB code: 6HRQ. Crystallized ligand and re-docked ligand poses are shown in cyan and magenta, respectively. Protein backbone is shown as a cartoon (white ribbon) and side chains of key amino acid residues in the active sites are shown as white sticks. The catalytic zinc ion and conserved water molecule are shown as orange and red spheres, respectively.

Docking in protein structure PDB ID 6HRQ showed good re-docking results (Figure 3) as well as the best cross-docking results, where the docking poses of 21 out of 25 docked ligands showed an rmsd ≤ 2 Å (Table S1). Failure to reproduce the docking pose of some of the crystal structure could be attributed to (i) a different ZBG (e.g., 4CQF had a thiol ZBG while other structures had a benzhydroxamate ZBG) (ii) different compound/linker class (e.g., while we were focused on reproducing the binding pose of amide or amine meta-substituted benzhydroxamates linkers, PDB Codes: 4BZ7 and 4CQF possess linear and flexible linkers lacking the aromatic portion while 6HSH had a heteroaromatic linker with two rings connected (2-(piperidin-1-yl)pyrimidine)).

**Figure 4:**
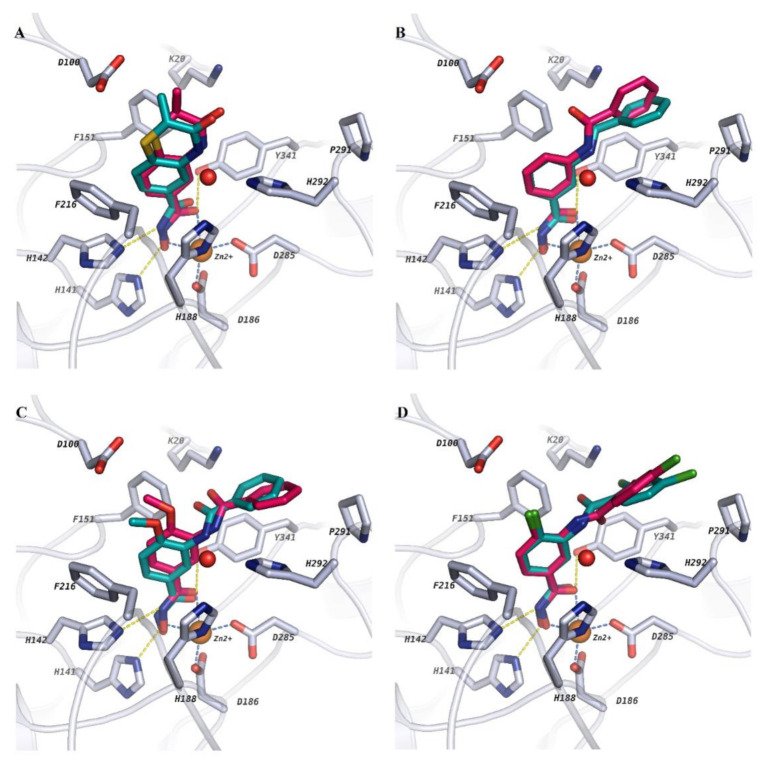
Comparing the docking poses derived from our docking protocol with the crystallized *sm*HDAC8 ligands. Co-crystallized ligands were taken from the corresponding PDB IDs by overlapping (**A**) PDB ID: 4BZ8, (**B**) PDB ID: 5FUE, (**C**) PDB ID: 6HT8 and (**D**) PDB ID: 6HU1. Co-crystallized ligands with their experimentally determined binding mode are shown in cyan while the docking pose of the ligands from our docking protocol in PDB ID: 6HRQ are shown in magenta. In the different pictures, protein backbones are shown as ribbons and side chains of key amino acid residues in the active sites are shown as white sticks. Catalytic zinc ion and conserved water molecule are shown as orange and red spheres, respectively.

In general, the chosen docking protocol could reproduce the experimentally determined binding mode of the already reported ligands with an rmsd < 2 Å (Figure 4). In all cases, the hydroxamate group (ZBG) of the docked ligands coordinates the zinc ion in a bidentate manner as well as showing other interactions (such as the hydrogen bonds between the ligands and His141, His142, Lys20, His292 and Tyr341 residues in addition to π–π staking interactions with residues lining the lysine binding tunnel and the side pocket) to stabilize the ligand–protein complex. Interestingly, based on our docking protocol, the selected poses were also top ranked using the standard precision (SP) scoring function of Glide in Schrödinger software. Hence, the top-ranked docking pose for each ligand was selected for further calculations after careful visual inspection. However, the correlation between the docking scores and the experimentally reported activities was poor (r^2^ ~ 0.009) (Model 1; Table 2; Table S1). Thus, the obtained docking poses were rescored by means of binding free energy calculations as discussed in the following section.

### 2.3. Analysis of BFE

Due to the low correlation (r^2^) observed between the Glide-SP docking score and the experimentally determined activities, we further evaluated the affinities of the ligands to *sm*HDAC8 using complex minimization and MM-PB/GBSA and QM/MM methods (using Amber software). The top scored docking poses from the molecular docking step were rescored either using a single frame (after short minimization steps) or several frames from the short MD simulations (1 ns). Different GB models, namely GB^HCT^ (igb = 1), GB^OBC^ (igb = 2), GB^OBC2^ (igb = 5), and GBn (igb = 8), as well as PB_mbondi (mbondi), PB_bondi (bondi) and PB_Parse (PARSE) were used to estimate the BFE. Moreover, the hybrid QM/MM–GBSA approach was also implemented, where the ligand and the catalytic Zn^2+^ ions were treated as the QM region using Parameterized Model number 3 (PM3) and Austin Model 1 (AM1) semi-emperical Hamilitonian theories in combination with GB1 solvation [55,56,57,58,59,60,61,62,63,64,65,66].

As mentioned in Section 3.3 and Section 3.4, models were generated using either the combination of terms summing up to the total energy term or the overall calculated energy. Furthermore, we tried to improve the quality of some of the models with the addition of a 2D-descriptor (Fractional positive polar van der Waals surface area) computed in MOE. The fractional positive polar van der Waals surface area (PEOE_VSA_FPPOS) was chosen amongst other descriptors to observe how the overall charge property of the molecules can contribute to explaining the observed activity of the molecules. In summary, 126 models were generated based on the docking score, complex minimization as well as the different MMGB-PB/SA and QM/MM methods/models used. The obtained correlation results using the different methods are shown in the SI (Table S2). In Table 2, selected correlation results are listed to give an overview on the performance of the different methods. In general, BFEs from MM-PBSA using the PB-bondi radii averagely outperformed the other methods in explaining the reported experimental activities.

### 2.4. Derived QSAR Models and Their Validation

As aforementioned, BFEs were computed using either a single frame from energy minimization or several frames from MD simulations. An application of the Partial least square (PLS) method was then applied to generate QSAR models based on the computationally estimated BFEs from the MM-PB/GBSA and QM/MM methods. For instance, in the MD simulation step, selected frames/snapshots (every fifth frame from the first frame for 1–50, 51–100 and 101–500 frames) were used to compute BFEs for the model development. The models were first constructed using various response variables (Y-variables) (1) the total calculated energy ∆G (2) the deltaG_gas_ and deltaG_sol_ values that sum up to the total energy. However, these models generated based on the computed BFEs did not show sufficient correlation with the observed biological activities. In order to improve the performance, we also investigated the effect of considering further computed descriptors in combination with the estimated BFEs to explain the reported biological activities. Thus, resulting models developed in this study were developed with or without the computed 2D descriptor. The developed QSAR models used in this study are exemplified in Table 2 (the entire list of models is presented in Table S2). Overall, we constructed 126 models using the BFEs calculated at different steps. These models were developed using several approaches (please refer to Section 2.3 and Section 3.3) so as to explore every means to explain variance in the observed activity of the molecules. Overall, models were judged based on their r^2^ and q^2^ values. For this study, better r^2^ and q^2^ values for the training set was observed with model 97 when compared to those from the initial “model 94”; both models generated using the PB-bondi radii. Model 97 was developed through the continuous improvement of model 94 (Table 2). The progress in the development of the topped model from each method utilized herein will be discussed in the following paragraphs with main focus on models 94 to 97 from MM-PBSA using the PB-bondi radii calculation.

We observed that all the models developed using MM-GBSA with GB^HCT^ (igb = 1), GB^OBC^ (igb = 2), GB^OBC2^ (igb = 5), and GBn (igb = 8) solvation models did not lead to satisfactory results and the computed BFEs were unable to explain the reported activity of the molecules in the training set. Just as observed in Table 2 above, the best model obtained using GB^HCT^ (igb = 1) calculations was model 2*3* which was designed using the computed contributions of deltaG_gas_ and deltaG_sol_ at the second energy minimization step and including the 2D descriptor PEOE_VSA_FPPOS (Equation (1)). This model showed a correlation coefficient (r^2^) of 0.51 and rmse of 0.3. However, further statistical validation of the model using the LOOCV approach led to very low q^2^ and higher qmse; thus, the discarding of the model. Meanwhile, all models developed using GB2, GB5 and GB8 showed an r^2^ < 0.5.

pIC_50__pred = 2.21599 − 0.05272 * deltaG_gas_ − 0.05911 * deltaG_sol_ + 12.20274 * PEOE_VSA_FPPOS (1)

Application of the MM-PBSA method (using PB-bondi radii) led to the stepwise development of a satisfactory model which showed good correlation with the biological data and good cross validation results. First, model 94 was developed based on the computed BFE from the MD step using every fifth frame from the first frame for frames 51–100. Exploration of the model revealed that its predictive performance was only moderate, as it could explain only about 45% variance of the reported biological activity (r^2^ of 0.45) while having an rmse of 0.35 (Table 2; Equation (2)). Therefore, we analyzed the molecular properties of the studied inhibitors and calculated a variety of molecular descriptors and include them in the QSAR models. We identified PEOE_VSA_FPPOS; a 2D descriptor that improved the QSAR model (Model 95).

pIC_50__pred = 3.98023 − 0.03888 * ∆G (2)

**Figure 5:**
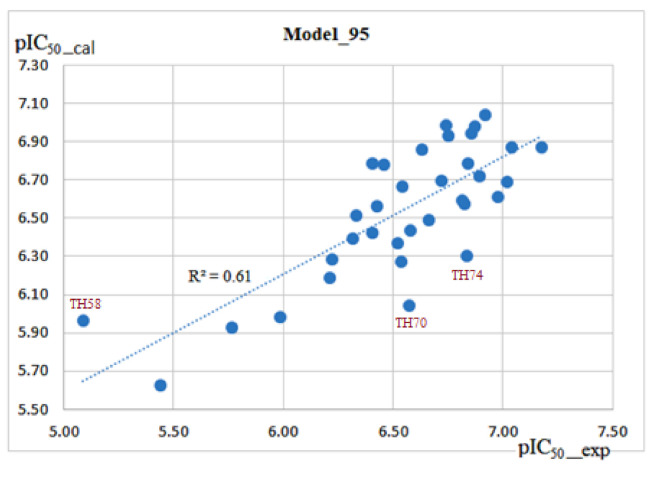
Correlation plot between the experimental pIC_50_ values (X-axis) and the calculated pIC_50_ values (Y-axis) for the training set molecules (blue points) using model 95.

Improvement of model 94 by including PEOE_VSA_FPPOS as a 2D descriptor led to model 95 (Equation (3)) which could better explain the correlation between the experimental and calculated activities. The r^2^ value was observed to increase from 0.45 (in model 94) to 0.61 (in model 95). In addition to the increased r^2^ values, q^2^ also increased from 0.36 to 0.53, 0.39 to 0.55 and 0.38 to 0.54 for LOOCV, Leave-3-out-CV, and 3fold-CV validations, respectively. This model was also considered to be more reliable because the rmse values were generally at acceptable limits (Figure 5).

pIC_50__pred = 3.31705 − 0.03592 * ∆G + 10.69909 * PEOE_VSA_FPPOS (3)

**Figure 6:**
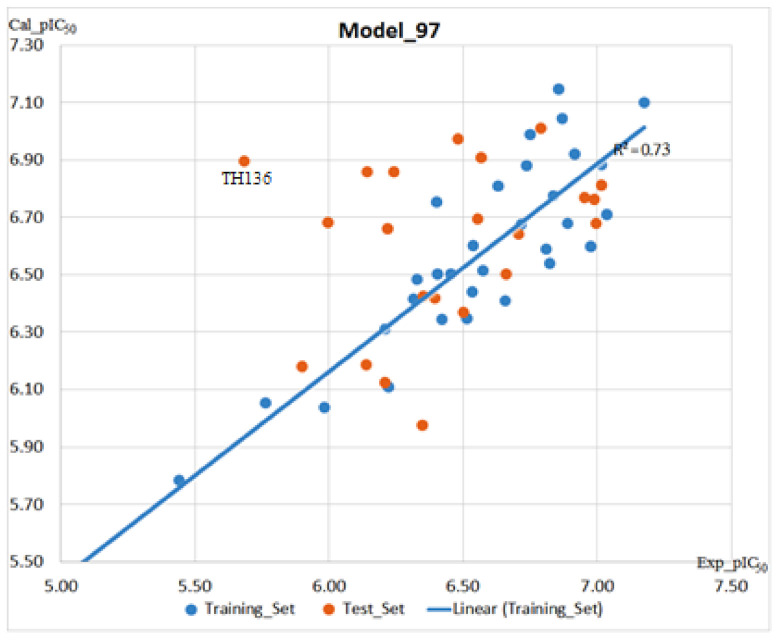
Correlation plot between the experimental pIC_50_ values (X-axis) and the calculated pIC_50_ values (Y-axis) for the training set molecules (blue points) using model 97 as well as the distribution of the newly designed set of molecules (orange points) along the linear regression line.

Analyzing Model 95 detected three outliers based on their calculated Z-score (Z-score > 2, compounds **TH58**, **TH70** and **TH74**) and removal of these compounds led to the development of Model 97 (Equation (4), Figure 6) with a significant difference in the r^2^, q^2^, rmse and qmse values when compared with model 95. Z-score in brief represents the absolute difference between the experimentally reported value and the predicted value base on the model in question divided by the square-root of the mean square error of the dataset. Z-scores were calculated for the molecules in this study using the QSAR module of MOE and molecules with Z-score greater than two were considered outliers. In addition to the statistical Z_score that was used to select molecules that were deemed as outliers, compounds **TH58** and **TH70** were further considered outliers based on the following observations/analyses. From another point of view, the computed 2D-descriptors to analyze the chemical space of the molecules could also be used to explain why **TH58** was an outlier. For instance, based on the chemical space analysis, **TH58** occupied a distant chemical space when compared to other molecules within the training set and it was thus not strange having it in the list of outliers. **TH70** on the other hand, although structurally similar to molecules of scaffold B, had the N-atom of the linker portion of the molecule replaced with an O-atom. This difference in atom type brought about observable changes in docking pose for compound **TH70** when compared with a structurally related compound (**TH28**). The docking pose of compound **TH70** showed that some important interactions including the hydrogen bond interaction observed between the docked molecules with His292 and Lys20 were missing. Additionally, a slight change in the coordinate of the aromatic linker was observed. The resulting model could explain ~73% variation of the reported experimental activity (Figure 6). For this reason, model 97 was also considered for further investigation and prediction of novel designed *sm*HDAC8 inhibitors.

pIC_50__pred = 3.45878 − 0.03231 * ∆G + 11.84528 * PEOE_VSA_FPPOS (4)

### 2.5. Evaluation of Novel Designed smHDAC8 Inhibitors

**Figure 7:**
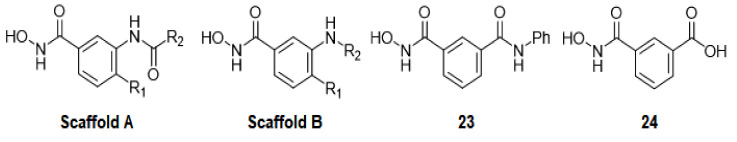
Chemical structures of novel *sm*HDAC8 inhibitors used as external test set.

To further evaluate the reliability and predictive power of the best performing models, the best models were evaluated on a set of newly developed inhibitors (Figure 7, Table 3). Based on the obtained biological data of the previously described molecules in the training set, we utilized a structure-based approach in order to design further benzhydroxamate derivatives in an attempt to optimize their activity against *sm*HDAC8. Generally, the scaffolds (scaffold A and B) were maintained (except for an inverse amide derivative, compound **23**) while the substituents at the para- (R_1_) and meta- (R_2_) positions were altered. The maintained scaffolds have well established interactions with important amino acids such as Tyr304, His292, His140, His141, Lys20 as well as a bidentated coordination to the catalytic zinc ion; as seen in solved *sm*HDAC8 crystal structures. For instance, starting from the lead compounds **TH61** and **TH39** including several derivatives which bear an additional alkoxy substituent at the 2- and 2,4-positions of the phenyl capping group were designed, with the goal of better addressing the hydrophobic side pocket. This improved Van der Waals interactions led to increased activity as seen when moving from **TH66** to **TH92**. This observation led to the exploration of several hydrophobic substituents including the effects of bicyclic and tricyclic substituents. Docking pose prediction for such compounds showed that they could fit within the receptor active site while maintaining the necessary interactions from the scaffolds used. Thus, derivatives which have bulkier aromatic substituents at the 3- and 4-positions of the phenyl capping group, such as the biphenyl and phenoxyphenyl derivatives (**TH77** and **TH95**) were further modified by adding different substituents at the m-position of the benzhydroxamte moiety. Compound **TH60**, possessing a quinolyl moiety as a capping group, was another inhibitor within the training set which showed highly potent inhibition of *sm*HDAC8. Hence, further derivatives with bi- and tricyclic capping groups, such as compounds **2**, **6**, **7**, **18**, **19** and **20**, were designed and synthesized (details on the synthesis, crystallization and biological testing will be published elsewhere).

**Figure 8:**
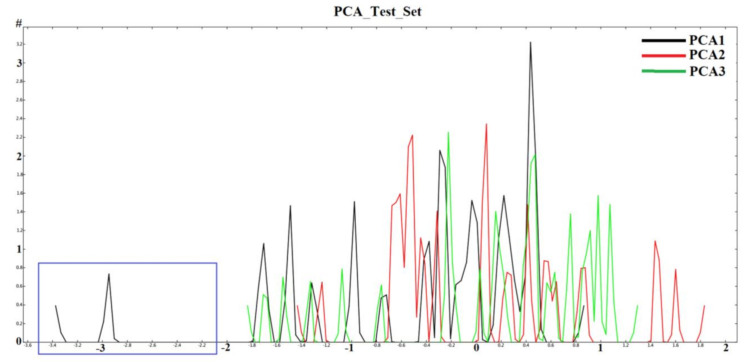
2D plots to visualize the variation of the three most important computed principal components for the test set (Blue box represent the outlier; compound **24**).

**Figure 9:**
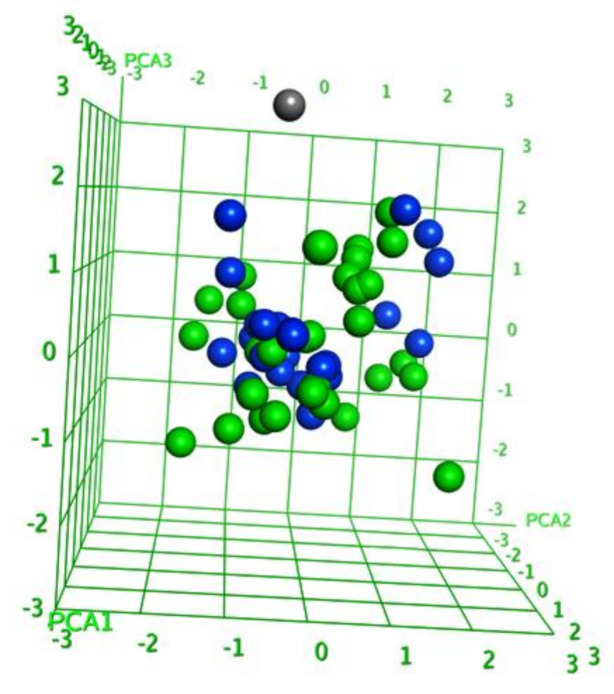
3D visualization of the first three PCAs to compare the chemical space occupied by training set (green balls) and newly designed molecules (blue balls) while the outlier (compound **24**) from the newly designed set is shown as the grey ball.

Analysis of the chemical space of the designed (test set) molecules in a similar way as for the training set revealed that the designed inhibitor **24** distanced itself from the remaining molecules (Figure 7 while the 2D and 3D distribution of the molecules can be seen in Figure 8 and Figure 9). Figure 9 shows that the computed properties of this molecule do not fall in line with the properties of the molecules designed based on the scaffolds (scaffold A and B) of choice for this study. To further visualize the position of the molecules used in this study, we performed extra graphical representation as seen in Figure 9. With the exception of compound **24** in the newly designed/external set, all the molecules were homogeneously distributed within the PCA space. Thus, the molecules used in this study generally occupied a similar chemical space. In addition to this observation, we could also explain the position of compound **24** as an outlier, based on the fact that this compound uniquely has a carboxylate substituent at the meta-position of the benzhydroxamate scaffold (Figure 7 and Figure 9) which plays a role in deviating its computed properties from the rest of the molecules.

In order to predict the activity of the newly designed (test set) molecules, docking and BFE calculations were also performed following the same protocols reported for the training set. Additionally, due to the good performance of Models 95, 96 and 97 (judging by the observed r^2^ and q^2^ values between experimental and predicted pIC_50_ values from the training set molecules; for predictions from Models 95 and 96, see Table S3), the three models were considered for the final prediction of activities of the newly designed molecules. We assessed the predictive quality of the selected models by calculating the difference between the experimentally determined and predicted pIC_50_ values. The IC_50_ values of the newly designed and synthesized compounds were measured using a similar procedure as described in previous publications [49,67]. The observed experimental activity was in the sub-micromolar to lower-micromolar range (Table 3). The IC_50_ values were subsequently converted to pIC_50_ for further evaluations. For the vast majority of the newly designed set of compounds, the absolute difference between the experimental and predicted pIC_50_ values was <0.7 log unit (which is less than 1000 nm) when considering all selected models. The low residual value between the experimentally measured activities and the predicted activities indicate that the models generated have good predictive ability.

**Figure 10:**
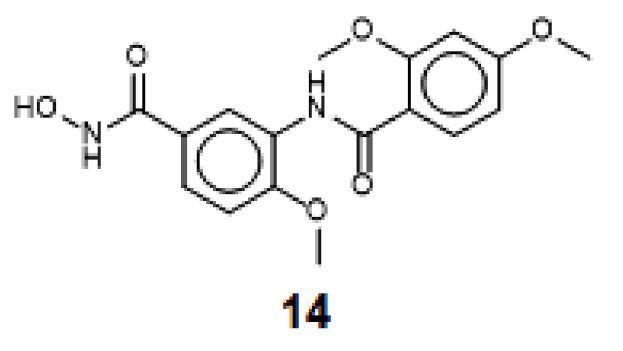
Structure of the poorly predicted molecule by *Model 97*.

**Figure 11:**
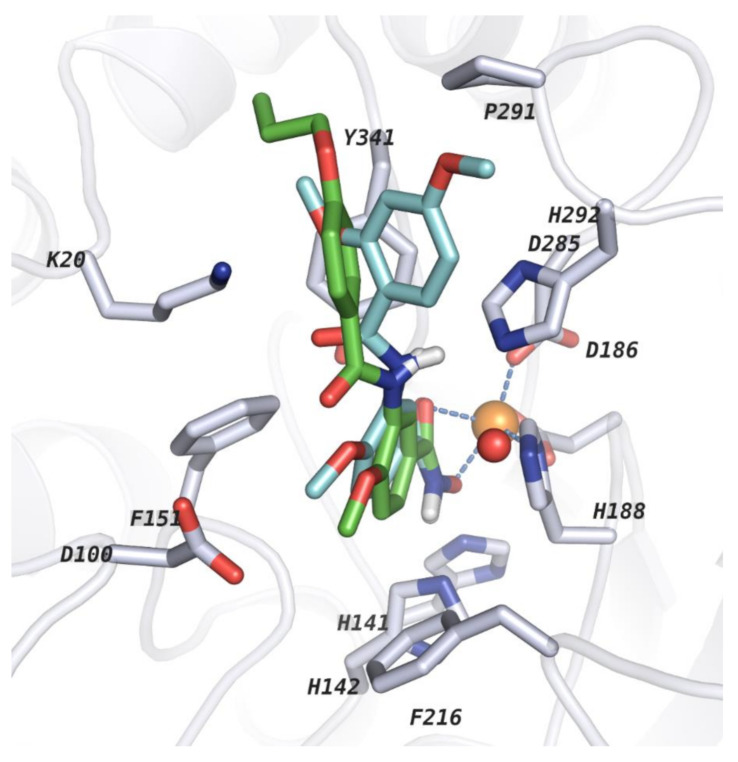
Comparison of the topped docking pose for the poorly predicted molecule (compound **14**; cyan) with a closely related molecule (compound **12**; green). Protein backbone is shown as ribbon and side chains of key amino acid residues in the active sites are shown as white sticks. Catalytic zinc ion and conserved water molecule are shown as orange and red spheres, respectively.

However, all top models failed to rightly predict the activity of one out of the 25 newly designed molecules (compound **14**; Figure 10, respectively), which showed weak experimental activity but was predicted to be highly active. Though there are no significant features setting this molecule apart from the rest of the molecules that were rightly predicted, it may be noted that this molecule possesses a capping group that could adopt different docking poses (Figure 11). Several docking poses were suggested for this molecule, with observable shift in coordinates of the atoms encompassing the capping group. Conversely, a detail look into the computed BFE and the different contributions summing-up to the total energy did not also show any abnormality. Thus, the model just failed to rightly predict this molecule, for reasons that are still to be identified. Looking at the residual between the average value of experimentally reported pIC_50_ of the training set that was used in generating model 97 and the predicted activity for each compound showed that ~99% of the molecules were rightly predicted. The distribution of the molecules along the regression line (Figure 6) confirmed that the predictions generally had deviations less than 400 nm.

## 3. Materials and Methods

### 3.1. Dataset Source, Preparation and Analysis

#### 3.1.1. Dataset

A set of 34 previously reported *sm*HDAC8-inhibitors with their *sm*HDAC8 IC_50_ values were used as the training set for this study (Figure 1) [54]. All compounds were synthesized as reported [54], and the in vitro inhibitory activities were determined using an enzymatic assay. The measured IC_50_ values were converted to pIC_50_ values for the QSAR study.

#### 3.1.2. Calculation of Molecular Descriptors and Dataset Diversity Analysis

Descriptors used in this work were calculated using MOE version 2016.08 [68]. Several 2D structural molecular descriptors for the training set and the newly designed series of benzhydroxamate derivatives were calculated. The calculated descriptors were further analyzed to ensure that there is no correlation between the descriptors using QuSAR-Contingency (a statistical application in MOE), and the resulting descriptors were submitted for further utilization in this work (Table 4). To investigate the chemical space coverage (diversity) of the molecules used in this study, we applied the PCA method implemented in the MOE package. The selected descriptors were transformed linearly using PCA [69,70]. This resulted in a new (smaller) table of descriptors that are uncorrelated and normalized (mean = 0 and variance = 1). Analysis of the variation of percentage counts and the 2- and 3-dimensional plots of the best three principal components (PCA1, PCA2 and PCA3) for all the datasets employed in this study was performed to check the diversity of the different sets.

### 3.2. Molecular Docking

#### 3.2.1. Ligand Preparation

The ligands used in this study were prepared using a similar approach as we previously reported, and which was successful in reproducing the X-ray structures of *sm*HDAC8–inhibitor complexes [52,71]. We started with the generation of 3D structures of the inhibitors using MOE 2016.08 [68]. The generated molecules were further processed using the “Wash” option implemented in MOE to deprotonate strong acids and protonate strong bases. The resulting dataset, containing the ligands only in their hydroxamate form, was subsequently prepared for docking using the LigPrep tool as implemented in Schrödinger’s software (version 2017-1) [72]. In this step, the generated ionization state obtained from MOE was kept; all possible tautomeric forms as well as stereoisomers were generated after which an initial structural energy minimization was performed using the integrated Optimized Potentials for Liquid Simulations 2005 (OPLS-2005) force field [73]. Finally, the ConfGen module also implemented in the Schrödinger’s software package was used to generate 50 conformers of the prepared molecules while allowing minimization of the output conformations [74,75].

#### 3.2.2. Protein Preparation

We used the same preparation protocol as in our earlier publications [50,52,54,71]. At the time of this work, 25 of the 28 published crystal structures for *sm*HDAC8 were used (we excluded PDB Codes: 6HSF and 6HSG because they had mutated residues (from the schistomal His-292 residue to the human MET-292 residue) while PDB Code: 4BZ5 was excluded because it lacked a crystallized ligand). All crystal structures of *sm*HDAC8 were imported into MOE from the PDB database (www.rcsb.org (Accessed 16 January 2020)) [68,76]. For each crystal structure, all water molecules (except the conserved water molecule (HOH744) within the catalytic pocket that was used for the docking procedures) were deleted. The Protein Preparation Wizard of Schrödinger software was subsequently used for further preparations of the protein structure [77,78]. At this juncture, bond orders were assigned and hydrogen atoms added. Additionally, the hydrogen bond network was optimized and the protonation states at pH 7.0 were predicted using the Epik-tool in Schrödinger [79,80]. Finally, a restrained energy minimization step (rmsd of the atom displacement for terminating the minimization was 0.3 Å) using the OPLS-2005 force field was performed on the system [73].

#### 3.2.3. Grid Generation and Docking

For the 25 prepared protein structures, a receptor grid for each protein structure was generated using the receptor grid preparation module implemented in Schrödinger. In this step, the center of the co-crystallized ligand for each crystal structure corresponded to the centroid of the grid box. Additionally, a metal constraint to the conserved catalytic zinc metal ion was included while keeping other options as default. The docking protocol using Glide in the standard precision mode (SP-score) was first validated by (1) redocking (its ability to reproduce the binding mode of a co-crystallized ligand with the binding site of its crystalized protein) and (2) cross-docking (the ability to rightly predict the binding mode of ligands from other *sm*HDAC8 crystal structures). The entire re-docking and cross docking results are provided as a supplementary file. The best generated protocol, with PDB Code: 6HRQ (being able to reproduce the crystallized ligands poses with low rmsd) was then used to dock the prepared training set ligands using Glide [81,82,83]. A total of 20 poses per ligand conformer were included in the post-docking minimization step. The top-ranked docking pose for each ligand was selected and submitted for protein–ligand interactions (BFE analysis).

### 3.3. Binding Free Energy (BFE) Calculations

The obtained top-ranked docking poses were subjected to BFE calculations using different radii sets (GB^HCT^ (igb = 1), GB^OBC^ (igb = 2), GB^OBC2^ (igb = 5), and GBn (igb = 8); Poison Boltzmann mbondi, bondi and PARSE), with the Tip3P solvation method and 12-6-4LJ ion model [84,85,86,87,88]. Preparation of ligands, protein, complexes and calculation methods employed in this step of the study are similar to those previously reported and are briefly described in the following sub-sections [89].

#### 3.3.1. Ligand and Ligand–Protein Complex Preparation

Antechamber package in AMBER16 was used to prepare the top-ranked docking pose for each ligand using the semi-empirical Austin Model 1 (AM1) with Bond Charge Correction (BCC) (AM1-BCC) [90]. In this process, assignment of atom type, bond type, judging of the atomic equivalence, generation of residue topology file, as well as finding missing force field parameters and supplying reasonable and similar substitutes with the parmchk function of amber were done. The tleap module in AMBER was then used to prepare the various protein–ligand complexes.

Hydrogen atoms were added to all amino acid residues assuming a normal ionization state for all ionizable residues. The Duan et al. (2003) force field (ff03.r1) and general amber force field (gaff) were used for protein and ligand optimization, respectively [91,92,93]. TIP3P water solvation model was used to solvate the systems in an octahedral box, leaving at least 10 Å between the solute atoms and the borders of the box [85]. We also applied 12-6-4LJ ionic models for the zinc ion [94]. Additionally, 15 Na^+^ ions were required to neutralize each complex system.

The GPU-accelerated version (the accelerated version of the graphical processing unit) of the pmemd (pmemd.cuda) script in AMBER16 was used to run our pre-BFE calculations [95,96]. First, the solvated complexes were energy minimized using a 4000-cycle minimization in two steps; (2000 cycles of steepest descent followed by 2000 cycles of a conjugate gradient) with restraints (force constant of 10 kcal*mol^−1^*Å^−2^) on the protein, zinc and ligand atoms, while the solvent molecules and counter-ions were free. A further 4000 cycles (2000 cycles of steepest descent followed by 2000 cycles of a conjugate gradient) minimization without restraints of the entire systems was performed to remove any steric clash in the initial geometry of the protein. Subsequently, the system was heated over 100 ps from 0 to 300 K while restraining the solute (force constant of 10 kcal*mol^−1^*Å^−2^) and the density then evaluated. An equilibration step over a period of 200 ps was launched to equilibrate the systems before the MD step. Afterwards, a 1-ns MD simulation with a time step of 2 fs was applied using the Particle Mesh Ewald method [97,98]. During the equilibration and MD steps, the systems were kept at constant temperature of 300 K regulated with a Langevin thermostat with a collision frequency of 2 ps^−1^ and pressure of 1 bar maintained using isotropic position scaling with a relaxation time of 2 ps. The SHAKE algorithm was applied to constrain all bonds involving hydrogens. Frames were written every 0.002 ns. Finally, a third energy minimization process using 4000 cycles (2000 cycles of steepest descent followed by 2000 cycles of a conjugate gradient) of the free systems after the MD step was performed. Snapshots for analysis were written every 2 fs and the CPPTRAJ program inbuilt in AMBER16 was used to analyze the systems. A nonbonded cutoff distance of 10 Å was used in all steps.

#### 3.3.2. MM-PB/GBSA and QM/MM Based BFE Prediction

To estimate the respective binding free energies of the docked molecules against *sm*HDAC8, molecular mechanics Poisson–Boltzmann/Generalized-Born Surface Area (MM-PB/GBSA) and the merged Quantum Mechanics/Molecular Mechanics (QM/MM) calculations were performed using the AMBER16 simulation package [90]. The MMPBSA.py script which utilizes the trajectory of complex only to create ensemble average of both the receptor and the ligand was used to estimate the BFE for each ligand–protein complex [99]. Various MM-PB/GBSA methods using different GB models, namely GB^HCT^ (igb = 1), GB^OBC^ (igb = 2), GB^OBC2^ (igb = 5), and GBn (igb = 8), as well as the MM-PBSA method with different bond radii (bondi and PARSE) were utilized [55,56,57,58,59,60,61,62,63,64,65,66]. Additionally, the semi-empirical methods including Parameterized Model number 3 (PM3) and Austin Model 1 (AM1) in combination with the GB1 solvation model were used in the hybrid QM/MM step to estimate the interaction energy between the receptor and ligand [61,62,100,101]. In this QM/MM approach, QM potentials were applied on the ligand and catalytic Zn^2+^ ion (constituting part of the receptor) while we apply MM force fields to remain part of the ligand–protein complex system. For the different methods listed, BFEs were computed at various stages of our protocol as described in Section 3.3.1, i.e., after each energy minimization step (Emin1 output structure of first minimization step used, Emin2: output frame of second minimization step used, and Emin3: output structure of minimization step of MD simulation last frame) and over different intervals of the MD simulation step (MD_p1; 1 to 50, MD_p2: 51 to 100 and MD_p3: 101 to 500 frames).

Generally, correctly predicting binding free energies of compounds to their target assist in directing the synthesis of new and promising compounds towards a particular path. Interestingly, several methods have been proposed and some are being applied to computationally predict the relative binding affinities of small molecules to their target protein. In this study, the MM-PB/GBSA and the QM/MM were used to estimate the BFE which takes into account changes in the gas-phase energy, solvation free energy and configurational entropy upon complex formation. The predicted BFE is estimated from the contributions obtained from the deltaG_gas_ and deltaG_sol_ (with additionally the estimated Self-Consistent-Field Energy (ESCF) for QM/MM) components as shown in Equations (5) and (6) for MM-PB/GBSA and QM/MM, respectively [102,103,104,105,106,107,108].

(5) $$\Delta G={\mathrm{deltaG}}_{\mathrm{gas}}+{\mathrm{deltaG}}_{\mathrm{sol}}$$

(6) $$\Delta G={\mathrm{deltaG}}_{\mathrm{gas}}+{\mathrm{deltaG}}_{\mathrm{sol}}+\mathrm{ESCF}$$

The energy contributions are further broken into different terms/components; van der Waals energy (VDWAALS) and Electrostatic energy (EEL) for G_gas_ phase and polar solvation energy (EGB) and Non-polar solvation energy (ESURF) for GB calculations to obtain G_sol_ (or polar solvation energy (EPB) and Non-polar solvation energy (ENPOLAR) for PB). Thus, Equations (5) and (6) can then be expanded to Equations (7) and (8), respectively.

(7) $$\Delta G=\mathrm{VDWAALS}+\mathrm{EEL}+\mathrm{EGB}\left(\mathrm{EPB}\right)+\mathrm{ESURF}\left(\mathrm{ENPOLAR}\right)$$

(8) $$\Delta G=\mathrm{VDWAALS}+\mathrm{EEL}+\mathrm{EGB}\left(\mathrm{EPB}\right)+\mathrm{ESURF}\left(\mathrm{ENPOLAR}\right)+\mathrm{ESCF}$$

### 3.4. Quantitative Structure–Activity Relationship (QSAR) Model Development and Selection

For the development of the models, a training set of 34 molecules whose selection is described above (see Section 3.1.1 and Section 3.2.1) was used. Correlating the biological activities with the estimated BFE and calculated theoretical descriptors of the ligands, QSAR-models were generated and studied using the Partial Least Square (PLS) method implemented in MOE. It is important to note that the quality of QSAR models depends on the selection of molecules in the dataset, distribution of the property being evaluated (in this case biological activity) and the chosen descriptors. In general parlance, models in the form of equations that provide a relationship between the dependent variable (usually biological activity) and independent variable (computed descriptors) are constructed using regression methods. In the current study, QSAR models were developed using either the different terms summing-up to the overall BFE or the total computed BFE (see Section 3.3.2. above). Several validation methods were used to validate the models developed using the training set. Models were selected based on the quality of their regression coefficient (r^2^), root mean squared error (rmse), leave-one-out cross-validated explained variance (q^2^) and crossed-root mean squared error (qmse). Acceptable models were based on high r^2^ (>0.5) and q^2^ (>0.5) values with low rmse (≤0.3) and qmse (≤0.3) values. In general, models derived using QM/MM methods were not selected due to low r^2^ and q^2^ values. Further robust verification and internal validation of the selected models from the MM-PB/GBSA calculations were done using leave-three-molecules-out (Leave-3out) as well as a 3fold cross-validation [109,110]. Final models employed in this study were models that could maintain a low qmse (<0.3) and q^2^ (>0.5) values upon the robust validations. The final models were then prospectively validated through the design, synthesis and biological validation of novel set of molecules (referred to as “test set” in this paper).

### 3.5. Test Set Prediction

To explore the reliability of the selected QSAR models, we further evaluated the predictive power of the models on an external set of molecules, which were synthesized and evaluated for their inhibitory activity on *sm*HDAC8. The predictive power of the developed models was evaluated using the experimentally determined IC_50_ values which were converted to pIC_50_ (x-variable) and the computed BFE (y-variable). It is worth noting that the predictions were all done using the MOE software [68].

## 4. Conclusions

Although several efforts have been put in place to eradicate schistosomiasis (bilharzia); the disease continues to ravage hundreds of millions of humans in underprivileged communities. Reports on drug resistance against praziquantel (the current drug of choice) stands as a factor forcing researchers to design and develop novel anti-schistosomial compounds, especially those capable of interfering with the parasite’s epigenome. In our previous publications [52,53,54], we showed that *sm*HDAC8 inhibitors could be used to target the pathogen. In the current study, we explored different methods to develop several QSAR models to explain the variation of the observed biological activity using 34 previously reported *sm*HDAC8 inhibitors from our research group as our training set [54]. Initially, we performed docking studies to test the ability of the utilized docking settings to reproduce the binding pose of the already crystallized molecules and suggest the most probable binding pose for molecules with no crystal structures based on confirmed interactions for this chemical scaffold. However, the inability of the Glide-SP docking score to explain the variation of the reported experimental activity of the molecules prompted us to use post-processing methods (BFE calculations) to re-score the docking pose. Further attempts using predicted BFEs from different GB models, namely GB^HCT^ (igb = 1), GB^OBC^ (igb = 2), GB^OBC2^ (igb = 5), and GBn (igb = 8), as well as PB_mbondi (mbondi), PB_bondi (bondi), PB_Parse (PARSE) using our docked poses led to the development of several QSAR models. The QSAR model (model 94) consisting of the computed BFE at the MD step using 10 frames (every fifth frame from the first frame for frames 51–100) of the PB_bondi radii calculation was selected for investigation. Further improvement of this model using a 2D descriptor and/or the removal of outliers led to models 95–97 (with correlation coefficients of 0.61, 0.62 and 0.73, respectively) that were better in ranking and explaining the variation of the observed biological activity in the training set. Moreover, the predictive strength of models 95–97 were further validated on a set of newly designed and synthesized molecules which occupy a similar chemical space as the molecules in the training set. The predicted biological activities of the newly designed molecules using the models were in good accordance with the experimentally determined activity of the molecules, proving that our models possess reliable predictive power. Therefore, the models completely fulfill the requirements for the suggestion of new *sm*HDAC8 inhibitors. We intend to use the information with regard to the binding energy of proposed *sm*HDAC8 inhibitors to continue the chemical optimization in order to identify new *sm*HDAC8 inhibitors.

The prediction of HDAC selectivity based on calculated interaction energy is still challenging and was not addressed in the current manuscript. It requires high quality data, also for selectivity, obtained for a large series of compounds. Often selectivity is only measured between individual isoforms and only for a few highly promising compounds, as we did in the current study of *sm*HDAC8 inhibitors. We observed good selectivity of the compounds for *sm*HDAC8 in comparison to human HDAC1 and 6, but since the activity of most of the inhibitors is rather low on these enzymes we did not determine an IC_50_ value for hsHDAC1/6 (only for a few compounds). Therefore, the data set included here is not suitable to do a quantitative modelling of HDAC selectivity. In case of the selectivity between human and *sm*HDAC8 we obtained only low to moderate selectivity so far, which means the range of selectivity is not high enough for quantitative modelling. Therefore, novel more selective *sm*/*hs*HDAC8 inhibitors have to be developed that might be used to develop predictive BFE/QSAR models for *sm*/*hs*HDAC8 selectivity.

## Figures and Tables

**Table 1:** Training set molecules and their respective experimental pIC_50_ and SP-Docking Sores.

Name	Compound Code *	Scaffold	R_1_	R_2_	*sm*HDAC8 IC_50_ (nM)	pIC_50_-*sm*HDAC8	GLIDE SP_Score
TH100	13r	A	methoxy	4-ethoxyphenyl	305 ± 35	6.52	−7.14
TH101	13s	A	methoxy	benzyl	183 ± 39	6.74	−7.51
TH104	13z	A	chloro	2,4-dichlorophenyl	191 ± 17	6.72	−7.20
TH31	13a	A	hydrogen	phenyl	468 ± 79	6.33	−8.52
TH33	13b	A	methyl	phenyl	154 ± 0.03	6.81	−7.48
TH39	13c	A	methoxy	phenyl	135 ±0.03	6.87	−7.60
TH60	13k	A	methyl	2-quinolyl	96 ± 13	7.02	−7.04
TH61	13e	A	chloro	phenyl	67 ± 10	7.17	−7.31
TH65	13l	A	methoxy	4-biphenyl	293 ±35	6.53	−7.35
TH66	13h	A	ethoxy	phenyl	129 ± 8	6.89	−7.43
TH67	13i	A	propoxy	phenyl	267 ± 49	6.57	−7.51
TH68	13n	A	methoxy	4-chlorophenyl	146 ± 4	6.84	−7.34
TH69	13m	A	methoxy	4-methoxyphenyl	106 ± 18	6.97	−7.31
TH74	13t	A	chloro	4-methoxyphenyl	147 ± 5	6.83	−7.65
TH75	13f	A	bromo	phenyl	150 ± 9	6.82	−7.41
TH76	13d	A	fluoro	phenyl	178 ± 8	6.75	−7.28
TH83	13j	A	isopropoxy	phenyl	220 ± 56	6.66	−8.58
TH85	13o	A	methoxy	2-chlorophenyl	351 ± 16	6.45	−7.22
TH86	13p	A	methoxy	2,4-dichlorophenyl	122 ± 19	6.92	−7.98
TH92	13za	A	ethoxy	4-biphenyl	92 ± 26	7.04	−8.58
TH93	13x	A	chloro	4-chlorophenyl	235 ± 10	6.63	−7.36
TH94	13g	A	trifluoromethyl	phenyl	140 ± 8	6.86	−7.10
TH95	13q	A	methoxy	3-biphenyl	290 ± 20	6.54	−7.28
TH96	13y	A	chloro	4-nitropheny	394 ± 50	6.40	−7.06
TH77	13u	A	chloro	3-benzyloxyphenyl	378 ± 45	6.42	−9.49
TH78	13v	A	chloro	3-phenoxyphenyl	396 ± 43	6.40	−9.55
TH81	13w	A	chloro	4-phenoxyphenyl	620 ± 70	6.21	−8.85
TH58	14a	-	-	-	8210 ± 1300	5.09	−7.51
TH36	10c	-	-	-	1722 ± 910	5.76	−7.41
TH70	15a	-	-	-	268 ± 21	6.57	−7.34
TH71	16a	-	-	-	485 ± 158	6.31	−7.58
TH28	10a	B	hydrogen	benzyl	1040 ± 250	5.98	−7.15
TH32	10b	B	hydrogen	cyclohexyl	3630 ± 620	5.44	−6.80
TH35	10e	B	methyl	cyclohexyl	600 ± 196	6.22	−7.16

* Compound code from the original publication (Heimburg et al. [54]).

**Table 2:** Summary of statistical interpretation of the selected models.

Model Number	Method	Frame	Number of Molecules	lm		LOOCV		Leave_3out CV		3fold CV		Outlier
				r^2^	RMSE	q^2^	QMSE	q^2^	QMSE	q^2^	QMSE	
1			34	0.01	0.44	-	-	-	-	-	-	
6	AM1/GB1	Emin2	34	0.41	0.34	0.30	0.37	-	-	-	-	
7	AM1/GB1	Emin2	34	0.51	0.31	0.38	0.35	-	-	-	-	
22	GB^HCT^ (igb = 1)	Emin2	34	0.31	0.37	0.13	0.42	-	-	-	-	
23	GB^HCT^ (igb = 1)	Emin2	34	0.51	0.31	0.32	0.36	-	-	-	-	
33	GB^OBC^ (igb = 2)	Emin1	33	0.30	0.37	0.19	0.40	-	-	-	-	
34	GB^OBC^ (igb = 2)	Emin1	34	0.46	0.32	0.33	0.36	-	-	-	-	
48	GB^OBC^ (igb = 5)	Emin1	34	0.31	0.36	0.20	0.40	-	-	-	-	
49	GB^OBC^ (igb = 5)	Emin1	34	0.47	0.32	0.34	0.36	-	-	-	-	
65	GBn (igb = 8)	Emin2	34	0.19	0.40	0.02	0.48	-	-	-	-	
77	PB-mbondi	Emin2	34	0.27	0.38	0.08	0.43	-	-	-	-	
94	PB-bondi	MD51-100	34	0.45	0.33	0.36	0.35	0.39	0.36	0.38	0.36	
95	PB-bondi	MD51-100	34	0.61	0.27	0.53	0.30	0.55	0.30	0.54	0.31	
96	PB-bondi	MD51-100	33	0.62	0.22	0.53	0.25	0.56	0.25	0.54	0.26	TH58
97	PB-bondi	MD51-100	31	0.73	0.19	0.66	0.22	0.70	0.21	0.69	0.22	TH58, TH70, TH74
105	PB-PARSE	Emin2	34	0.17	0.40	0.01	0.46	-	-	-	-	
117	PM3/GB1	Emin2	34	0.37	0.35	0.26	0.38	-	-	-	-	
118	PM3/GB1	Emin2	34	0.52	0.31	0.41	0.34	-	-	-	-	

lm: linear model, LOOCV: Leave-one out cross validation, Leave_3out CV: Leave-3-out cross validation, 3fold CV: 3fold cross validation, PM3/GB1: Parameterized Model number 3 in combination with GB1 solvation, AM1/GB1: Austin Model 1 in combination with GB1 solvation, Emin2: single frame after the second energy minimization step, Emin1: single frame after the first energy minimization step, MD51-100: every fifth frame from the first frame frames for 51–100 during the MD simulation run.

**Table 3:** Chemical structure and prediction of test set compounds.

Compound Number	Compoud Code	Scaffold	R1	R2	*sm*HDAC8_IC_50_ nM-	pIC_50__exp	pIC_50__pred	Res ^a^ (Exp_pIC_50_-Pred_pIC_50_)	Res ^b^ Avg_Exp_pIC_50_-Pred_pIC_50_	Glide SP_Score
**1**	AT_T4	A	methoxy	9*H*-fluoren-1-yl	163 ± 17	6.79	7.01	−0.22	−0.44	−9.20
**2**	SD14	A	methoxy	3-methyl-1,2,3,4-tetrahydro-ɣ-carbolin-8-yl	197 ± 19	6.71	6.64	0.06	−0.07	−9.24
**3**	TH112	A	ethoxy	2,4-dichlorophenyl	103 ± 7	6.99	6.77	0.22	−0.20	−8.99
**4**	TH117	A	chloro	4-biphenyl	404 ± 90	6.39	6.42	−0.03	0.15	−8.69
**5**	TH119	A	methanethiolyl	4-biphenyl	101 ± 7	6.99	6.68	0.31	−0.11	−8.47
**6**	TH120	A	chloro	benzo[b]thien-7-yl	97 ± 16	7.01	6.81	0.20	−0.24	−8.90
**7**	TH125	A	methoxy	1H-benzo[d]imidazol-2-yl	575 ±72	6.24	6.86	−0.62	−0.29	−9.48
**8**	TH127	A	methoxy	3-benzyloxyphenyl	605 ± 68	6.22	6.66	−0.44	−0.09	−9.23
**9**	TH128	A	methyl	3-benzyloxyphenyl	447 ± 31	6.35	6.43	−0.08	0.14	−8.63
**10**	TH132	A	methoxy	2-chloro-4-biphenyl	101 ± 77	7.00	6.68	0.31	−0.11	−8.71
**11**	TH133	A	chloro	2-chloro-4-biphenyl	112 ± 11	6.95	6.77	0.18	−0.20	−8.35
**12**	TH134	A	methoxy	4-propoxyphenyl	729 ± 86	6.14	6.19	−0.05	0.38	−8.51
**13**	TH135	A	methoxy	4-isopropoxyphenyl	725 ± 52	6.14	6.86	−0.72	−0.29	−8.76
**14**	TH136	A	methoxy	2,4-dimethoxyphenyl	2078 ± 273	5.68	6.90	−1.22	−0.33	−9.29
**15**	TH137	A	chloro	2,4-dimethoxyphenyl	220 ± 13	6.66	6.51	0.15	0.06	−9.22
**16**	TH138	A	methoxy	2-chloro-4-(4-fluorophenyl)phenyl	318 ± 19	6.50	6.37	0.13	0.20	−8.99
**17**	TH139	A	chloro	2-chloro-4-(4-fluorophenyl)phenyl	281 ± 37	6.55	6.70	−0.14	−0.13	−8.66
**18**	TH142	A	methoxy	quinolin-8-yl	332 ± 51	6.48	6.98	−0.50	−0.41	−9.84
**19**	TH143	A	methoxy	4-dibenzofuranyl	271 ± 30	6.57	6.91	−0.34	−0.34	−9.76
**20**	TH156	B	methoxy	4-dibenzofuranyl	451 ± 90	6.35	5.98	0.37	0.59	−9.42
**21**	TH34	B	methyl	benzyl	1260 ± 170	5.90	6.18	−0.28	0.39	−9.15
**22**	TH42	B	methoxy	benzyl	620 ± 0	6.21	6.13	0.08	0.44	−9.74
**23**	TH97	-	-	-	220 ± 67	6.66	6.77	−0.11	−0.26	−8.13
**24**	TH98	-	-	-	1590 ± 190	5.80	6.49	−0.69	0.08	−9.59

^a^ Difference between the experimental pIC_50_ and the predicted pIC_50_; ^b^ difference between the average pIC_50_ (excluding outliers) and the predicted pIC_50_.pIC_50__Exp is the pIC_50_ obtained from the reported experimental activity while Pred_pIC_50_ is the predicted pIC_50_ and corresponds to each model used.

**Table 4:** List of selected 2D descriptors used to analyze the chemical space of molecules employed in this study.

Notation	Molecular Descriptors
a_heavy	Number of heavy atoms
b_1rotN	Number of rotatable single bonds. Conjugated single bonds are not included
b_single	Number of single bonds (including implicit hydrogens). Aromatic bonds are not considered to be single bonds.
lip_acc	The number of O and N atoms.
lip_don	The number of OH and NH atoms.
mr	Molecular refractivity (including implicit hydrogens).
PEOE_VSA_POL	The number of OH and NH atoms.
TPSA	Polar surface area (Å2) calculated using group contributions to approximate the polar surface area from connection table information only.
h_logD	The octanol/water distribution coefficient at pH 7.
PEOE_VSA_FPPOS *	Fractional positive polar van der Waals surface area.

* Included in some of the developed QSAR models for quality improvement.

## Data Availability

Not applicable.

## Supplementary Materials

Table S1: Summary of re-docking and crossed-docking rmsd between the atomic coordinates of the co-crystalized ligands pose and the docked pose based on the different *sm*HDAC8 crystalized proteins used in this study. Table S2: Summary of generated moldels. (Terms are described after the table). Table S3: summary of prediction results based on Models 95 and 96.
