# Supplementary material for: Binding Free Energy (BFE) Calculations and Quantitative Structure–Activity Relationship (QSAR) Analysis of Schistosoma mansoni Histone Deacetylase 8 (smHDAC8) Inhibitors
Source: Molecules. 2021 Apr 28;26(9):2584. doi: 10.3390/molecules26092584 (PMC8125515; doi:10.3390/molecules26092584)
Supplement: Supplementary file 1 [file molecules-26-02584-s001.zip › molecules-1185691-supplementary.pdf]

Table SI_1: Summary of redocking and crossed-docking rmsd between the atomic coordinates of the co-crystalized ligands pose and the docked pose based for the different *sm*HDAC8 crystalized proteins used in this study.

PDB-ID.Chain	4BZ6	4BZ7	4BZ8	4BZ9	4CQF	5FUE-	6FU1	6GX3	6GXA	6GXU	6GXW	6HQY	6HRQ
4BZ7.A	9.332	4.3122	8.4564	6.2498	1.4452	10.5651	3.7347	9.237	4.5097	4.3047	11.6499	9.7755	4.1727
4BZ8.A	1.9422	2.6006	0.4615	2.842	2.6747	1.1467	2.7661	1.2743	2.7208	1.9431	1.8886	1.6547	1.2012
4BZ9.A	2.0847	2.0288	2.6946	0.3396	2.7801	2.1921	3.1253	1.8144	1.3388	2.1466	1.8798	2.2516	1.7569
4CQF.A	4.9007	4.1145	7.1393	2.883	6.9373	6.2036	4.3287	5.8476	4.2973	6.4933	5.8749	5.2396	5.4873
5FUE.C	2.1622	6.7069	4.0167	4.5767	2.3976	0.2285	5.38	0.8602	1.0938	0.6308	0.4061	1.3743	1.1051
6FU1.A	3.6687	4.3572	2.864	3.0835	2.2008	1.9365	3.0918	2.3943	1.9777	2.3025	2.1027	3.6532	1.2999
6GX3.A	0.5777	0.7548	0.835	1.9492	2.4252	0.3278	0.8507	0.2251	1.7416	0.8281	0.7766	0.8298	0.2711
6GXA.A	1.3769	2.234	1.7334	1.0604	2.3359	2.3426	2.4712	1.4553	0.4793	0.6372	2.0627	1.2037	1.5241
6GXU.A	1.3501	3.3411	3.8291	4.492	3.7483	1.1129	3.6936	1.6125	0.7961	0.4852	4.2575	0.5783	1.9285
6GXW.A	2.1889	2.4824	4.1894	4.7054	2.6549	2.3426	4.3176	0.9205	2.3324	2.6544	2.6926	1.9319	1.9171
6HQY.A	1.0926	2.589	2.0821	4.6798	3.2144	2.516	7.7507	3.0304	1.2488	1.1617	1.3191	1.0973	1.5272
6HRQ.A	3.5725	3.0107	3.1511	6.6396	3.237	3.7134	9.1757	1.1086	1.2374	3.7371	3.9295	3.5973	1.2334
6HSH.A	4.7782	5.9525	6.012	6.5674	7.3283	7.0334	5.9234	6.7578	5.9959	5.3594	7.0996	5.5554	6.9159
6HSZ.A	1.6523	1.7632	1.5933	4.7527	2.0691	0.4933	4.9007	1.0589	1.1799	0.838	0.4535	1.3768	0.7972
6HT8.A	1.4837	3.3922	3.2984	4.7837	2.0626	1.1378	4.8869	0.8283	1.2026	0.7567	1.1452	0.84	0.8562
6HTG.A	1.3729	1.5782	3.3922	4.7658	2.0342	0.6535	4.9101	1.2454	1.4802	0.6503	0.4209	1.656	0.7281
6HTH.A	2.0912	10.2397	5.7554	8.2105	9.7153	0.9478	10.2968	9.3249	10.2201	2.1194	1.3112	2.2352	1.3666
6HTI.A	0.8893	7.4348	5.2577	4.8621	1.8751	0.529	6.1181	1.0582	2.4365	2.1022	0.2775	2.1073	2.3637
6HTT.A	1.4622	1.3879	4.7083	4.5048	1.4894	0.9366	5.0682	0.7295	1.2387	1.2724	0.3749	0.9844	0.7058
6HTZ.A	1.7298	4.3216	0.8592	5.8732	1.64	2.1535	4.4801	1.8051	2.1218	1.6341	2.6759	1.979	2.0899
6HU0.A	2.075	1.3073	1.0971	4.7861	1.529	1.7382	1.5618	1.1484	1.4551	1.5764	0.1958	1.9665	0.7085
6HU1.A	2.102	6.7757	1.1067	4.7702	1.4049	0.3369	4.9664	1.3534	0.5683	1.5986	0.3296	2.0174	1.5453
6HU2.A	1.1195	5.9628	1.7928	3.7928	1.4959	1.1001	1.7313	1.6632	3.1334	1.6106	1.5749	1.489	1.5037
6HU3.A	1.6811	4.0606	1.676	5.7871	4.3484	1.1511	4.7526	2.122	1.2805	1.6576	2.1264	0.9933	1.6091

Table SI_1 continues:

PDB-ID.Chain	6HSF	6HSG	6HSH	6HSZ	6HT8	6HTG	6HTH	6HTI	6HTT	6HTZ	6HU0	6HU1	6HU2	6HU3
4BZ7.A	4.3259	11.7683	8.5947	4.542	7.0551	7.2655	8.5568	11.4642	11.9157	7.2795	8.6283	6.9584	6.6607	9.0698
4BZ8.A	2.3995	2.5231	0.4889	1.2148	1.2483	1.1465	1.2112	0.9469	1.5686	0.9471	0.8695	1.4058	1.3091	1.2325
4BZ9.A	1.7223	1.9405	2.6786	1.6724	1.6868	1.6681	1.5903	1.7097	1.7625	1.5835	2.2557	1.7022	1.7505	1.7573
4CQF.A	4.7954	6.0275	7.2997	6.1045	5.7546	5.8918	5.9252	5.9031	6.4936	6.0347	6.0145	6.1087	3.2012	6.0738
5FUE.C	1.098	1.4228	3.776	1.2374	1.1966	0.7651	1.1542	1.1541	0.9666	0.6276	0.5128	1.1433	0.334	1.745
6FU1.A	3.4126	1.4356	2.8504	3.3438	3.1604	3.5533	3.493	3.4472	1.612	3.3221	3.3627	3.4544	4.2121	3.8013
6GX3.A	0.5179	0.2629	2.4233	0.314	0.4391	0.3678	0.258	0.2309	0.4224	0.486	0.3187	0.4229	0.3873	0.2472
6GXA.A	0.4841	1.5584	2.4351	1.2183	1.3081	1.236	1.4895	1.4687	1.5669	1.2311	1.4541	1.3298	2.1755	1.4392
6GXU.A	1.0603	1.879	4.0798	1.1255	0.8696	1.0953	0.9033	1.0742	1.6149	1.0982	1.616	0.9457	0.9246	1.7186
6GXW.A	2.1414	2.4673	4.3247	2.2055	2.6379	2.6369	1.9889	2.5127	2.234	1.8033	2.5228	1.9491	2.6938	0.9207
6HQY.A	0.4563	2.6082	3.5936	1.2731	1.2478	1.2364	1.2251	0.8772	1.4845	0.6936	1.4132	1.2287	2.7205	2.879
6HRQ.A	2.9867	3.6883	3.1347	3.5488	3.6454	3.6861	3.7329	3.7964	3.7322	3.6766	1.0958	3.6857	3.7021	4.2912
6HSH.A	5.2334	3.7783	2.1169	5.0552	6.791	6.7121	6.8086	5.264	4.8627	6.0086	6.6144	5.3291	6.7415	5.8004
6HSZ.A	1.0275	1.4203	3.5569	0.2039	1.1557	0.4074	0.2448	0.1853	0.4433	0.3608	0.3503	1.1081	1.1028	0.3914
6HT8.A	1.1606	1.1082	3.3789	1.1087	0.3455	1.1005	0.2833	0.2418	0.9039	1.108	1.1226	0.2873	0.4163	1.0896
6HTG.A	1.343	1.4882	3.6202	0.5683	1.2034	1.3065	1.2207	1.1936	0.8411	0.6426	0.6158	0.6038	0.5835	1.1637
6HTH.A	1.431	1.5726	6.2781	1.2579	0.872	0.8991	1.2599	0.94	1.2859	1.2451	0.4833	0.8708	0.9802	1.4562
6HTI.A	1.8805	1.0324	1.7887	2.3245	2.3616	0.5084	2.1992	0.2136	0.2198	2.3161	0.4989	2.3152	0.3807	0.3011
6HTT.A	1.2791	0.7953	4.9248	1.234	1.2087	0.6695	1.2256	0.7739	1.114	1.2942	0.4459	1.232	1.1775	0.7149
6HTZ.A	1.8456	1.775	1.7363	1.0981	1.2377	0.8571	0.8534	0.7563	1.4654	1.3305	0.7963	1.6517	1.7004	1.2219
6HU0.A	1.6417	0.9399	2.8095	1.5929	0.3368	0.4675	1.5395	0.2074	1.6905	1.587	1.5895	0.2689	0.2541	0.6136
6HU1.A	1.94	1.0048	3.4199	1.6207	1.5903	0.4473	0.2553	0.2101	1.5318	1.6397	0.3958	0.2116	0.3659	1.1334
6HU2.A	1.7851	1.4728	1.6978	1.4639	1.4345	1.6859	1.219	1.5269	1.1034	1.4226	0.9396	1.7415	1.9088	2.7986
6HU3.A	2.5645	0.6011	4.9322	1.3152	1.178	1.3191	1.9757	0.9486	4.9418	0.9405	1.1937	1.2948	1.1364	1.606

The colour shadings from dark green through light green, yellow, light red to thick red shows how well the co-crystalized ligand coordinates were reproduce to how poor they were redocked respectively.

Table SI_2: Summary of generated moldels. (Terms are described after the table)

Model_Number	**Model-composition**	**lm**	**LOOCV**			**leave_3out CV**		**3fold CV**		**Presence of 2D-Descriptor**	**Outlier**
		**R-squared**	**RMSE**	**Q-squared**	**QMSE**	**Q-squared**	**QMSE**	**Q-squared**	**QMSE**		
1	docking_score	0.01	0.44	0.53	0.52	-	-	-	-	-	
2	AM1/GB1_Emin3_1Des	0.17	0.40	0.05	0.44	-	-	-	-	-	
3	AM1/GB1_Emin3_3Des	0.26	0.38	0.08	0.43	-	-	-	-	-	
4	AM1/GB1_Emin1_1Des	0.26	0.38	0.15	0.41	-	-	-	-	-	
5	AM1/GB1_Emin1_3Des	0.28	0.37	0.07	0.47	-	-	-	-	-	
6	AM1/GB1_Emin2_1Des	0.41	0.34	0.30	0.37	-	-	-	-	-	
7	AM1/GB1_Emin2_1Des	0.51	0.31	0.38	0.35	-	-	-	-	YES	
8	AM1/GB1_Emin2_3Des	0.44	0.33	0.25	0.39	-	-	-	-	-	
9	AM1/GB1_Emin2_3Des	0.44	0.33	0.25	0.39	-	-	-	-	YES	
10	AM1/GB1_MD101to500_1Des	0.27	0.38	0.15	0.41	-	-	-	-	-	
11	AM1/GB1_MD101to500_3Des	0.29	0.37	0.13	0.42	-	-	-	-	-	
12	AM1/GB1_MD1to50_1Des	0.21	0.39	0.10	0.42	-	-	-	-	-	
13	AM1/GB1_MD1to50_3Des	0.30	0.37	0.13	0.42	-	-	-	-	-	
14	AM1/GB1_MD1to50_3Des	0.30	0.37	0.13	0.42	-	-	-	-	YES	
15	AM1/GB1_MD51to100_1Des	0.18	0.40	0.08	0.43	-	-	-	-	-	
16	AM1/GB1_MD51to100_3Des	0.26	0.38	0.11	0.42	-	-	-	-	-	
17	GB^{HCT} (igb=1)_Emin3_1Des	0.02	0.44	0.08	0.47	-	-	-	-	-	
18	GB^{HCT} (igb=1)_Emin3_2Des	0.06	0.43	0.02	0.47	-	-	-	-	-	
19	GB^{HCT} (igb=1)_Emin1_1Des	0.27	0.38	0.14	0.41	-	-	-	-	-	
20	GB^{HCT} (igb=1)_Emin1_2Des	0.29	0.37	0.14	0.42	-	-	-	-	-	
21	GB^{HCT} (igb=1)_Emin2_1Des	0.27	0.38	0.13	0.42	-	-	-	-	-	
22	GB^{HCT} (igb=1)_Emin2_2Des	0.31	0.37	0.13	0.42	-	-	-	-	-	
23	GB^{HCT} (igb=1)_Emin2_2Des	0.51	0.31	0.32	0.36	-	-	-	-	YES	
24	GB^{HCT} (igb=1)_MD101to500_1Des	0.15	0.41	0.03	0.45	-	-	-	-	-	
25	GBHCT (igb=1)_MD101to500_2Des	0.17	0.40	0.02	0.45	-	-	-	-	-	
26	GB^{HCT} (igb=1)_MD1to50_1Des	0.14	0.41	0.03	0.44	-	-	-	-	-	
27	GB^{HCT} (igb=1)_MD1to50_2Des	0.19	0.40	0.05	0.44	-	-	-	-	-	

28	GB^{HCT} (igb=1)_MD1to50_4Des	0.28	0.38	0.04	0.48	-	-	-	-	-
29	GB^{HCT} (igb=1)_MD51to100_1Des	0.13	0.41	0.02	0.45	-	-	-	-	-
30	GB^{HCT} (igb=1)_MD51to100_2Des	0.16	0.40	0.04	0.44	-	-	-	-	-
31	GB^{OBC} (igb=2)_Emin3_1Des	0.21	0.39	0.11	0.42	-	-	-	-	-
32	GB^{OBC} (igb=2)_Emin3_2Des	0.21	0.39	0.06	0.44	-	-	-	-	-
33	GB^{OBC} (igb=2)_Emin1_1Des	0.30	0.37	0.19	0.40	-	-	-	-	-
34	GB^{OBC} (igb=2)_Emin1_1Des	0.46	0.32	0.33	0.36	-	-	-	-	YES
35	GB^{OBC} (igb=2)_Emin1_2Des	0.31	0.37	0.17	0.41	-	-	-	-	-
36	GB^{OBC} (igb=2)_Emin1_2Des	0.31	0.37	0.17	0.41	-	-	-	-	YES
37	GB^{OBC} (igb=2)_Emin2_1Des	0.22	0.39	0.09	0.43	-	-	-	-	-
38	GB^{OBC} (igb=2)_Emin2_2Des	0.22	0.39	0.07	0.44	-	-	-	-	-
39	GB^{OBC} (igb=2)_MD101to500_1Des	0.17	0.40	0.07	0.43	-	-	-	-	-
40	GB^{OBC} (igb=2)_MD101to500_2Des	0.18	0.40	0.05	0.44	-	-	-	-	-
41	GB^{OBC} (igb=2)_MD1to50_1Des	0.10	0.42	0.02	0.45	-	-	-	-	-
42	GB^{OBC} (igb=2)_MD1to50_2Des	0.14	0.41	0.02	0.45	-	-	-	-	-
43	GB^{OBC} (igb=2)_MD1to50_4Des	0.22	0.39	0.03	0.45	-	-	-	-	-
44	GB^{OBC} (igb=2)_MD51to100_1Des	0.17	0.40	0.04	0.44	-	-	-	-	-
45	GB^{OBC} (igb=2)_MD51to100_2Des	0.17	0.40	0.03	0.45	-	-	-	-	-
46	GB^{OBC2} (igb=5)_Emin3_1Des	0.20	0.39	0.10	0.42	-	-	-	-	-
47	GB^{OBC2} (igb=5)_Emin3_2Des	0.20	0.39	0.05	0.44	-	-	-	-	-
48	GB^{OBC2} (igb=5)_Emin1_1Des	0.31	0.36	0.20	0.40	-	-	-	-	-
49	GB^{OBC2} (igb=5)_Emin1_1Des	0.47	0.32	0.34	0.36	-	-	-	-	YES
50	GB^{OBC2} (igb=5)_Emin1_2Des	0.34	0.36	0.19	0.40	-	-	-	-	-
51	GB^{OBC2} (igb=5)_Emin1_2Des	0.34	0.36	0.19	0.40	-	-	-	-	YES
52	GB^{OBC2} (igb=5)_Emin2_1Des	0.19	0.40	0.06	0.44	-	-	-	-	-
53	GB^{OBC2} (igb=5)_Emin2_2Des	0.19	0.40	0.05	0.44	-	-	-	-	-
54	GB^{OBC2} (igb=5)_MD101to500_1Des	0.15	0.41	0.05	0.43	-	-	-	-	-
55	GB^{OBC2} (igb=5)_MD101to500_2Des	0.17	0.40	0.04	0.44	-	-	-	-	-
56	GB^{OBC2} (igb=5)_MD1to50_1Des	0.08	0.42	0.01	0.45	-	-	-	-	-
57	GB^{OBC2} (igb=5)_MD1to50_2Des	0.13	0.41	0.02	0.45	-	-	-	-	-
58	GB^{OBC2} (igb=5)_MD51to100_1Des	0.15	0.41	0.03	0.45	-	-	-	-	-

59	GB^{OBC2} (igb=5)_MD51to100_2Des	0.15	0.41	0.01	0.45	-	-	-	-	-
60	GBn (igb=8)_Emin3_1Des	0.05	0.43	0.00	0.46	-	-	-	-	-
61	GBn (igb=8)_Emin3_2Des	0.07	0.42	0.00	0.46	-	-	-	-	-
62	GBn (igb=8)_Emin1_1Des	0.01	0.44	0.14	0.46	-	-	-	-	-
63	GBn (igb=8)_Emin1_2Des	0.07	0.43	0.00	0.46	-	-	-	-	-
64	GBn (igb=8)_Emin2_1Des	0.02	0.44	0.03	0.46	-	-	-	-	-
65	GBn (igb=8)_Emin2_2Des	0.19	0.40	0.02	0.48	-	-	-	-	-
66	GBn (igb=8)_MD101to500_1Des	0.04	0.43	0.04	0.47	-	-	-	-	-
67	GBn (igb=8)_MD101to500_2Des	0.07	0.43	0.01	0.47	-	-	-	-	-
68	GBn (igb=8)_MD1to50_1Des	0.12	0.41	0.03	0.44	-	-	-	-	-
69	GBn (igb=8)_MD1to50_2Des	0.17	0.40	0.05	0.44	-	-	-	-	-
70	GBn (igb=8)_MD51to100_1Des	0.15	0.41	0.05	0.44	-	-	-	-	-
71	GBn (igb=8)_MD51to100_2Des	0.19	0.40	0.06	0.44	-	-	-	-	-
72	PB_mbondi_Emin3_1Des	0.01	0.44	0.27	0.47	-	-	-	-	-
73	PB_mbondi_Emin3_2Des	0.01	0.44	0.41	0.48	-	-	-	-	-
74	PB_mbondi_Emin1_1Des	0.16	0.40	0.04	0.44	-	-	-	-	-
75	PB_mbondi_Emin1_2Des	0.19	0.40	0.04	0.45	-	-	-	-	-
76	PB_mbondi_Emin2_1Des	0.23	0.39	0.09	0.43	-	-	-	-	-
77	PB_mbondi_Emin2_2Des	0.27	0.38	0.08	0.43	-	-	-	-	-
78	PB_mbondi_MD101to500_1Des	0.07	0.42	0.00	0.47	-	-	-	-	-
79	PB_mbondi_MD101to500_2Des	0.12	0.41	0.00	0.48	-	-	-	-	-
80	PB_mbondi_MD1to50_1Des	0.07	0.43	0.00	0.46	-	-	-	-	-
81	PB_mbondi_MD1to50_2Des	0.11	0.42	0.00	0.46	-	-	-	-	-
82	PB_mbondi_MD51to100_1Des	0.02	0.44	0.05	0.47	-	-	-	-	-
83	PB_mbondi_MD51to100_2Des	0.02	0.44	0.13	0.49	-	-	-	-	-
84	PB_bondi_Emin3_1Des	0.19	0.40	0.09	0.42	-	-	-	-	-
85	PB_bondi_Emin3_2Des	0.20	0.39	0.05	0.44	-	-	-	-	-
86	PB_bondi_Emin1_1Des	0.15	0.41	0.03	0.44	-	-	-	-	-
87	PB_bondi_Emin1_2Des	0.17	0.40	0.02	0.45	-	-	-	-	-
88	PB_bondi_Emin2_1Des	0.20	0.39	0.07	0.43	-	-	-	-	-
89	PB_bondi_Emin2_2Des	0.21	0.39	0.03	0.45	-	-	-	-	-

90	PB_bondi_MD101to500_1Des	0.22	0.39	0.11	0.42	-	-	-	-	-	
91	PB_bondi_MD101to500_2Des	0.24	0.39	0.07	0.44	-	-	-	-	-	
92	PB_bondi_MD1to50_1Des	0.29	0.37	0.19	0.40	-	-	-	-	-	
93	PB_bondi_MD1to50_2Des	0.29	0.37	0.16	0.41	-	-	-	-	-	
94	PB_bondi_MD51to100_1Des	0.45	0.33	0.36	0.35	0.39	0.36	0.38	0.36	-	
95	**PB_bondi_MD51to100_1Des**	0.61	0.27	0.53	0.30	0.55	0.30	0.54	0.31	YES	
96	**PB_bondi_MD51to100_1Des**	0.62	0.22	0.53	0.25	0.56	0.25	0.54	0.26	YES	TH58
97	**PB_bondi_MD51to100_1Des**	0.73	0.19	0.66	0.22	0.70	0.21	0.69	0.22	YES	TH58, TH70, TH74
98	PB_bondi_MD51to100_2Des	0.46	0.32	0.32	0.37	-	-	-	-	-	
99	PB_bondi_MD51to100_2Des	0.46	0.32	0.32	0.37	-	-	-	-	YES	
100	PB_PARSE_Emin3_1Des	0.08	0.42	0.01	0.45	-	-	-	-	-	
101	PB_PARSE_Emin3_2Des	0.09	0.42	0.00	0.47	-	-	-	-	-	
102	PB_PARSE_Emin1_1Des	0.12	0.41	0.02	0.45	-	-	-	-	-	
103	PB_PARSE_Emin1_2Des	0.13	0.41	0.00	0.46	-	-	-	-	-	
104	PB_PARSE_Emin2_1Des	0.17	0.40	0.05	0.44	-	-	-	-	-	
105	PB_PARSE_Emin2_2Des	0.17	0.40	0.01	0.46	-	-	-	-	-	
106	PB_PARSE_MD101to500_1Des	0.12	0.41	0.03	0.44	-	-	-	-	-	
107	PB_PARSE_MD101to500_2Des	0.12	0.41	0.01	0.46	-	-	-	-	-	
108	PB_PARSE_MD1to50_1Des	0.02	0.44	0.02	0.45	-	-	-	-	-	
109	PB_PARSE_MD1to50_2Des	0.06	0.43	0.00	0.46	-	-	-	-	-	
110	PB_PARSE_MD51to100_1Des	0.05	0.43	0.00	0.45	-	-	-	-	-	
111	PB_PARSE_MD51to100_2Des	0.06	0.43	0.00	0.46	-	-	-	-	-	
112	PM3/GB1_Emin3_1Des	0.06	0.43	0.01	0.55	-	-	-	-	-	
113	PM3/GB1_Emin3_3Des	0.14	0.41	0.02	0.51	-	-	-	-	-	
114	PM3/GB1_Emin1_1Des	0.23	0.39	0.13	0.41	-	-	-	-	-	
115	PM3/GB1_Emin1_3Des	0.31	0.37	0.13	0.43	-	-	-	-	-	
116	PM3/GB1_Emin1_3Des	0.31	0.37	0.13	0.43	-	-	-	-	YES	
117	PM3/GB1_Emin2_1Des	0.37	0.35	0.26	0.38	-	-	-	-	-	
118	PM3/GB1_Emin2_1Des	0.52	0.31	0.41	0.34	-	-	-	-	YES	

119	PM3/GB1_Emin2_3Des	0.42	0.34	0.25	0.39	-	-	-	-	-
120	PM3/GB1_Emin2_3Des	0.42	0.34	0.26	0.38	-	-	-	-	YES
121	PM3/GB1_MD101to500_1Des	0.20	0.40	0.08	0.43	-	-	-	-	-
122	PM3/GB1_MD101to500_3Des	0.20	0.39	0.03	0.45	-	-	-	-	-
123	PM3/GB1_MD1to50_1Des	0.14	0.41	0.05	0.44	-	-	-	-	-
124	PM3/GB1_MD1to50_3Des	0.22	0.39	0.03	0.46	-	-	-	-	-
125	PM3/GB1_MD51to100_1Des	0.12	0.41	0.04	0.44	-	-	-	-	-
126	PM3/GB1_MD51to100_3Des	0.16	0.40	0.01	0.47	-	-	-	-	-

lm: linear model, **LOOCV**: Leave-one out cross validation, **Leave_3out CV**: Leave-3-out cross validation, **3fold CV**: 3fold cross validation, **PM3/GB1**: Parameterized Model number 3 in combination with GB1 solvation, **AM1/GB1**: Austin Model 1 in combination with GB1 solvation, **Emin3**: Single frame after the third energy minimization step, **Emin2**: Single frame after the second energy minimization step, **Emin1**: Single frame after the first energy minimization step, **MD1-50**: Every fifth frame from the first frame for 1 – 50 during the MD simulation run, **MD51-100**: Every fifth frame from the first frame for 51 – 100 during the MD simulation run. **MD101-500**: Every fifth frame from the first frame for 101 – 500 during the MD simulation run. **3Des**: the three energy terms summing up to the final energy (deltaG_gas, deltaG_sol and ESCF), **2Des**: the two energy terms summing up to the final energy (deltaG_gas and deltaG_sol), **1Des**: the final energy (deltaG)

Table_SI-3: summary of prediction results based on *Models 95* and *96*.

Compound Number	Name	smHDAC8 _nM-IC_{50}	pIC_{50}_ exp	$PRED_ Model_95	Residual Model_95	$PRED_ Model_96	Residual Model_96	GlideSP _DS
**1**	**AT_T4**	163 ± 17	6.79	7.03	-0.24	6.96	-0.17	-9.20
**2**	**SD14**	197 ± 19	6.71	6.73	-0.02	6.70	0.01	-9.24
**3**	**TH112**	103 ± 7	6.99	6.91	0.07	6.87	0.12	-8.99
**4**	**TH117**	404 ± 90	6.39	6.48	-0.09	6.51	-0.12	-8.69
**5**	**TH119**	101 ± 7	6.99	6.67	0.32	6.66	0.33	-8.47
**6**	**TH120**	97 ± 16	7.01	6.96	0.05	6.92	0.09	-8.90
**7**	**TH125**	575 ±72	6.24	6.84	-0.60	6.82	-0.58	-9.48
**8**	**TH127**	605 ± 68	6.22	7.02	-0.81	6.94	-0.72	-9.23
**9**	**TH128**	447 ± 31	6.35	6.82	-0.47	6.78	-0.43	-8.63
**10**	**TH132**	101 ± 77	7.00	6.64	0.36	6.63	0.37	-8.71
**11**	**TH133**	112 ± 11	6.95	6.87	0.08	6.82	0.13	-8.35
**12**	**TH134**	729 ± 86	6.14	6.22	-0.08	6.30	-0.16	-8.51
**13**	**TH135**	725 ± 52	6.14	6.96	-0.82	6.90	-0.76	-8.76
**14**	**TH136**	2078 ± 273	5.68	6.88	-1.19	6.83	-1.15	-9.29
**15**	**TH137**	220 ± 13	6.66	6.38	0.28	6.44	0.22	-9.22
**16**	**TH138**	318 ± 19	6.50	6.44	0.06	6.47	0.03	-8.99
**17**	**TH139**	281 ± 37	6.55	6.82	-0.27	6.78	-0.23	-8.66
**18**	**TH142**	332 ± 51	6.48	7.09	-0.61	7.01	-0.53	-9.84
**19**	**TH143**	271 ± 30	6.57	6.99	-0.42	6.92	-0.36	-9.76
**20**	**TH156**	451 ± 90	6.35	5.98	0.37	6.04	0.30	-9.42
**21**	**TH34**	1260 ± 170	5.90	6.27	-0.37	6.30	-0.40	-9.15
**22**	**TH42**	620 ± 0	6.21	6.35	-0.14	6.36	-0.15	-9.74
**23**	**TH97**	220 ± 67	6.66	6.71	-0.05	6.74	-0.09	-8.13
**24**	**TH98**	1590 ± 190	5.80	6.32	-0.52	6.38	-0.59	-9.59
